# Exercise Training Restores Cardiac MicroRNA-1 and MicroRNA-29c to Nonpathological Levels in Obese Rats

**DOI:** 10.1155/2017/1549014

**Published:** 2017-08-23

**Authors:** André C. Silveira, Tiago Fernandes, Úrsula P. R. Soci, João L. P. Gomes, Diego L. Barretti, Glória G. F. Mota, Carlos Eduardo Negrão, Edilamar M. Oliveira

**Affiliations:** ^1^Laboratory of the Biochemistry and Molecular Biology of Exercise, School of Physical Education and Sport, University of Sao Paulo, Sao Paulo, SP, Brazil; ^2^Heart Institute (InCor), Medical School, University of São Paulo, São Paulo, SP, Brazil

## Abstract

We previously reported that aerobic exercise training (AET) consisted of 10 weeks of 60-min swimming sessions, and 5 days/week AET counteracts CH in obesity. Here, we evaluated the role of microRNAs and their target genes that are involved in heart collagen deposition and calcium signaling, as well as the cardiac remodeling induced by AET in obese Zucker rats. Among the four experimental Zucker groups: control lean rats (LZR), control obese rats (OZR), trained lean rats (LZR + TR), and trained obese rats (OZR + TR), heart weight was greater in the OZR than in the LZR group due to increased cardiac intramuscular fat and collagen. AET seems to exert a protective role in normalizing the heart weight in the OZR + TR group. Cardiac microRNA-29c expression was decreased in OZR compared with the LZR group, paralleled by an increase in the collagen volumetric fraction (CVF). MicroRNA-1 expression was upregulated while the expression of its target gene NCX1 was decreased in OZR compared with the LZR group. Interestingly, AET restored cardiac microRNA-1 to nonpathological levels in the OZR-TR group. Our findings suggest that AET could be used as a nonpharmacological therapy for the reversal of pathological cardiac remodeling and cardiac dysfunction in obesity.

## 1. Introduction

Obesity results from a combination of excessive food energy intake, lack of physical activity, and genetic susceptibility [1–4]. Data from the World Health Organization (WHO) in 2014 showed that 1.9 billion people worldwide are overweight and 600 million are obese, causing 2.8 million deaths annually [5]. Obesity induces systemic inflammation and contributes to the development of atherosclerosis and cardiovascular diseases, which cooperate with the pathological cardiac hypertrophy (CH) phenotype [6, 7].

Cardiac remodeling induced by obesity is a compensatory adaptation to volume overload and/or continuous pressure imposed on the heart [8]. Studies in obese Zucker rats show an increase in left ventricular mass accompanied by pathological CH molecular markers such as *β*-myosin heavy chain (*β*-MHC), atrial natriuretic factor (ANF), *α*-skeletal actin; cardiac dysfunction; and ultimately, heart failure [8–10]. The diastolic dysfunction in obesity is induced both by increased collagen content and by damage to calcium signaling pathways mediated by proteins of intracellular calcium removal, such as SERCA-2a and the sodium/calcium exchanger NCX1 [11, 12].

Aerobic exercise training (AET) is a nonpharmacological strategy for preventing and treating obesity and cardiovascular disease [4, 13–16]. We have recently reported that AET reverses pathological cardiac remodeling in hypertensive and obese rats [4, 17, 18]. AET induces physiological CH by increasing the ratio of *α*/*β*-MHC and decreasing cardiac collagen content, improving ventricular compliance [19, 20]. Furthermore, AET leads to the restoration of normal calcium handling protein levels, potentially contributing to physiological cardiac hypertrophy and improved cardiac function [21].

MicroRNAs are regulators in various physiological and pathological processes, such as cardiac remodeling [22]. MicroRNAs are small endogenous RNAs that negatively regulate the expression of their target genes [23]. Previous data reported by our group showed that physiological CH induced by different amounts of AET is related to reduced cardiac collagen expression via elevated cardiac microRNA-29c levels in healthy rats [24]. In addition, Melo et al. [25] showed that AET restored the levels of microRNA-29c in infarcted rats, contributing to a reduction in cardiac collagen content. Interestingly, studies have shown the involvement of microRNAs in the regulation of calcium signaling pathways in the heart, indicating NCX1 as a target of microRNA-1 [26–28]. However, the effects of AET on cardiac microRNAs and cardiac remodeling in obesity are not fully established.

We investigated whether obesity increases cardiac collagen deposition and calcium handling proteins regulated by microRNAs and if AET restores these parameters, consequently contributing to the conversion of pathological into physiological CH in obesity.

## 2. Materials and Methods

### 2.1. Experimental Groups

Twenty male Zucker rats (20 weeks of age) were assigned to four groups (*n* = 5 each): control lean Zucker rats (LZR), trained lean Zucker rats (LZR + TR), control obese Zucker rats (OZR), and trained obese Zucker rats (OZR + TR). The animals were housed in cages, and food and water were provided ad libitum. The room temperature was 23°C, and an inverted 12 : 12 h dark-light cycle was maintained throughout the experiment.

All protocols and surgical procedures used were in accordance with the guidelines of the Brazilian College for Animal Experimentation and were approved by the Ethics Committee (1023/07) of the Biomedical Science Institute of the University of Sao Paulo.

### 2.2. Exercise Training Protocol

Swimming training was performed as described previously [4]. Animals were trained in a swimming apparatus specially designed to allow individual exercise training of rats in warm water at 30–32°C. Physical training consisted of swimming sessions of 60-min duration, five times a week, for 10 weeks, with 4% of body weight workload hold in tail [tail weight − % body weight (BW)]. All animals were weighed once a week and the workload was adjusted according to BW variations. LZR and OZR were placed in the swimming apparatus for 10 minutes twice a week without applying a workload. This protocol consists of a low/moderate intensity and long training period and is effective in promoting cardiovascular adaptations and increases in muscle oxidative capacity [19].

### 2.3. Tissue Harvesting

Twenty-four hours after the last training session, and after twelve hours of fasting, the rats were killed by quick decapitation. The tissues and tibia were harvested, the heart (H) was weighed, and carefully, the left ventricle (LV free wall plus septum) and right ventricle (RV) were dissected. Epididymal and retroperitoneal fats were also weighed and normalized by the tibial length (TL) of each animal. The tissues were frozen at −80°C until biochemical and molecular analysis was performed.

### 2.4. Cardiac Morphometric Analysis

For cardiomyocyte (CMO) diameter analysis, the LV was fixed in Tissue-Tekand frozen in liquid nitrogen. The tissues were then fixed in 6% formaldehyde, embedded in paraffin, cut into 10 *μ*m sections at the level of the papillary muscle in a cryostat (−20°C), and subsequently stained with hematoxylin and eosin for the visualization of cellular structures. Two randomly selected sections from each animal were visualized by light microscopy using an oil immersion objective with calibrated magnification (×400). CMOs with visible nuclei and intact cellular membranes were chosen for diameter determination. The width of individually isolated cardiomyocyte displayed on a viewing screen was manually traced across the middle of the nucleus with a digitizing pad, and the diameter was estimated using a computer-assisted image analysis system (Quantimet 520; Cambridge Instruments, Woburn, MA). For each animal, ~20 visual fields were analyzed. The results were expressed as micrometers (*μ*m).

The myocardial interstitial collagen volumetric fraction (CVF) was determined using the Picrosirius red prepared tissues, as reported previously [13]. In brief, 20 fields were selected from sections placed in a projection microscope (×200), and interstitial collagen was determined using a computer-assisted image analysis system (Quantimet 520; Cambridge Instruments). The CVF was calculated as the sum of all connective tissue areas divided by the sum of all muscle areas in all fields. Perivascular tissues (reparative fibrosis) were specifically excluded from this determination. The results were expressed as *μ*m for area.

LV intramuscular fat was determined using oil red staining. The tissues were cut into 7 *μ*m sections in a cryostat (−20°C) and fixed in 3.7% formalin for one hour. Subsequently, the tissues were washed with distilled water and then stained with a mixture of 12 ml working solution (500 mg oil red added to 100 ml of aqueous 60% triethyl phosphate [Fluka]) and 8 ml of deionized water for 30 seconds. The slides were assembled with the aid of glycerol [10% glycerol in 10 mM Tris-HCl, pH 8.5]. The area comprising intramuscular fat was determined using a computer-assisted image analysis system (Quantimet 520; Cambridge Instruments). The results were expressed as % of fiber area [29, 30].

### 2.5. Molecular Analysis

#### 2.5.1. mRNA and MicroRNA Quantification Using Real-Time PCR

The relative expression of COLIAI, COLIIIAI, ANF, *α*-MHC, *α*-actin skeletal, *β*-MHC, microRNA-1, microRNA-29a, microRNA-29b, and microRNA-29c was analyzed using real-time polymerase chain reactions (real-time PCR) as described previously [24]. Frozen heart samples (100 mg) were homogenized in Trizol (1 ml), and ribonucleic acid (RNA) was isolated according to the manufacturer's instructions (Invitrogen Life Technologies, Strathclyde, UK). Samples were quantified using a spectrophotometer at 260 nm and checked for integrity by EtBr-agarose gel electrophoresis. RNA was primed with 0.5 g/l oligo(dT) (12–18 bp) (Invitrogen Life Technologies) to generate the first strand of cDNA. Reverse transcription (RT) was performed using SuperScript II Reverse Transcriptase (Invitrogen Life Technologies). Primers were designed using Primer 3 software (http://frodo.wi.mit.edu/primer3/). DNA sequence was obtained from GenBank, and primers were designed in separate exons to distinguish PCR products derived from cDNA from those derived from genomic DNA contaminants on the basis of their size. The mRNA expression of type I/III collagen was assessed using oligonucleotide primers as follows: for COLIA, 5′-AgA gAg CAT gAC CgA Tgg A-3′ and 5′-gAggTT gCC AgT CTg TTg g-3′; for COLIIIA, 5′-AAg gTC CAC gAg gTg ACA A-3′ and 5′-Agg gCC Tgg ACT ACC AAC T-3′. Real-time quantification of the target genes was performed with a SYBRgreen PCR Master Mix (Applied Biosystems, PE, Foster City, CA) using an ABI PRISM 7500 Sequence Detection System (Applied Biosystems). The expression of cyclophilin A (5′-AAT gCT ggA CCA AAC ACA AA-3′ and 5′-CCT TCT TTC ACC TTC CCA AA-3′) was measured as a real-time PCR internal control. An aliquot of the real-time PCR reaction was used for 40-cycle PCR amplification in the presence of SYBRgreen fluorescent dye, according to the protocol provided by the manufacturer (Applied Biosystems, PE, Foster City, CA). The *α*-MHC and *β*-MHC mRNA expressions were assessed by oligonucleotide primers as follows: for *α*-MHC, 59-CGA GTC CCA GGT CAA CAA G-39 and 59-AGG CTC TTT CTG CTG GAC C-39); for *β*-MHC, 59-CAT CCC CAA TGA GAC GAA G-39 and 59-AGG CTC TTT CTG CTG GAC A-39; for *α*-skeletal actin, sense: 5′-ACC ACA GGC ATT GTT CTG GA-3′, antisense: 5′-TAA GGT AGT CAG TGA GGT CC-3′; and for ANF, sense: 5′-CTT CGG GGG TAG GAT TGA C-3′, antisense: 5′-CTT GGG ATC TTT TGC GAT CT-3′. The expression of cyclophilin A (59-AAT GCT GGA CCA AAC ACA AA-39 and 59-CCT TCT TTC ACC TTC CCA AA-39) was measured as an internal control for sample variation in real-time PCR reaction.

To accurately detect mature microRNAs, real-time PCR quantification was performed using primers for microRNA-1, microRNA-29a, microRNA-29b, and microRNA-29c (Life Technologies) with the TaqMan microRNA Assay protocol (Applied Biosystems, CA, USA). Samples were normalized by evaluating U6 expression. Each heart sample was analyzed in duplicate. Relative quantities of target gene and microRNA expression in the LZR, OZR, LZR + TR, and OZR + TR groups were compared after normalization using the expression values of internal controls [change in threshold cycle (ΔCT)]. Fold change was calculated using the differences in ΔCT values between the two samples (ΔΔCT) and the equation 2^−ΔΔCT^. The results are expressed as a percentage of the control value.

### 2.6. Western Blotting

The frozen hearts were thawed and homogenized in cell lysis buffer containing 100 mM Tris, 50 mM NaCl, 10 mM EDTA, 1% Triton X-100, and protease and phosphatase inhibitor cocktail [1 : 100; Sigma-Aldrich, MO, USA]. Insoluble heart tissues were removed by centrifugation at 3000*g*, 4°C, for 10 min. Samples were loaded and subjected to SDS-PAGE in 10% polyacrylamide gels. After electrophoresis, proteins were electrotransferred to nitrocellulose membranes (Amersham Biosciences, Piscataway, NJ). Equal loading of samples (50 *μ*g) and even transfer efficiency were monitored with the use of 0.5% Ponceau S staining of the blot membrane. The blot membrane was then incubated in a blocking buffer [5% nonfat dried milk, 10 mM Tris-HCl, pH 7.6, 150 mM NaCl, and 0.1% Tween 20] at room temperature and then with a polyclonal antibody directed against SERCA-2a (ab3625), PLB (ab86930), pPLB^ser16^ (ab15000), and NCX1 (ab2869) [1 : 1000; Abcam, Cambridge, United Kingdom] overnight at 4°C. Primary antibody binding was detected with the use of peroxidase-conjugated secondary antibodies, and enhanced chemiluminescence reagents (Amersham Biosciences, Piscataway, NJ) and detection were performed in a digitalizing unit (ChemiDoc; BioRad, CA, USA). The bands were quantified by ImageJ software (National Institute of Health, USA). GAPDH expression levels were used to normalize the results, which were expressed as a percentage of the control values as described previously [24].

### 2.7. Statistical Analysis

Results are represented as means ± standard error of the mean (SEM). Statistical analysis was performed using randomized two-way ANOVA. Tukey's post hoc test was used for individual comparisons between means when a significant change was observed with ANOVA. *p* ≤ 0.05 was considered as statistically significant.

## 3. Results

### 3.1. Adipose Tissue

**Figure 1:**
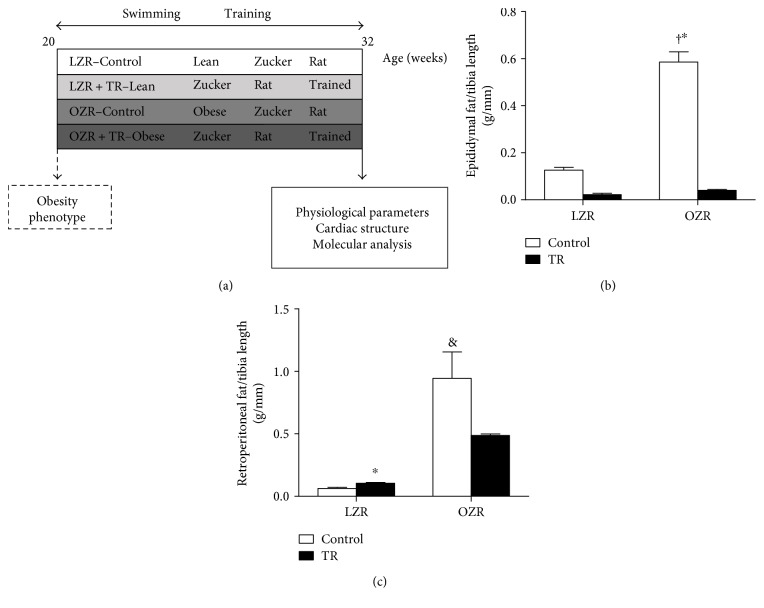
Effects of AET and obesity on epidydimal and retroperionetal fat. Schematic panel of study design (a). Content of retroperitoneal (b) and epidydimal fat (c) in LZR (control lean group), LZR + TR (trained lean group), OZR (control obese group), and OZR + TR (trained obese group). ^†^*p* < 0.01 versus LZR and LZR + TR, ^∗^*p* < 0.05 versus OZR + TR, and ^&^*p* < 0.001 versus LZR + TR.

We evaluated the effect of AET on body fat content in lean and obese groups after the training protocol (Figures 1(b) and 1(c)). As expected, the AET normalized epididymal fat content in the OZR + TR (0.04 ± 0.003 g/mm) groups compared with the control (0.126 ± 0.012 g/mm) and trained (0.022 ± 0.012 g/mm) LZR groups (Figure 1(b)). The epididymal fat content (Figure 1(b)) was decreased in OZR + TR (0.04 ± 0.007 g/mm) compared with OZR (0.58 ± 0.043 g/mm). In addition, AET was effective in reducing the epididymal fat content in LZR + TR (0.022 ± 0.006 g/mm) compared with the LZR group (0.126 ± 0.012 g/mm) (Figure 1(b)). The retroperitoneal fat content in the control OZR group was higher (0.94 ± 0.21 g/mm) than in the control LZR group (0.031 ± 0.25 g/mm; *p* < 0.0004) and LZR + TR (0.1 ± 0.007 g/mm; *p* < 0.0004) (Figure 1(c)).

### 3.2. Cardiac Hypertrophy

**Figure 2:**
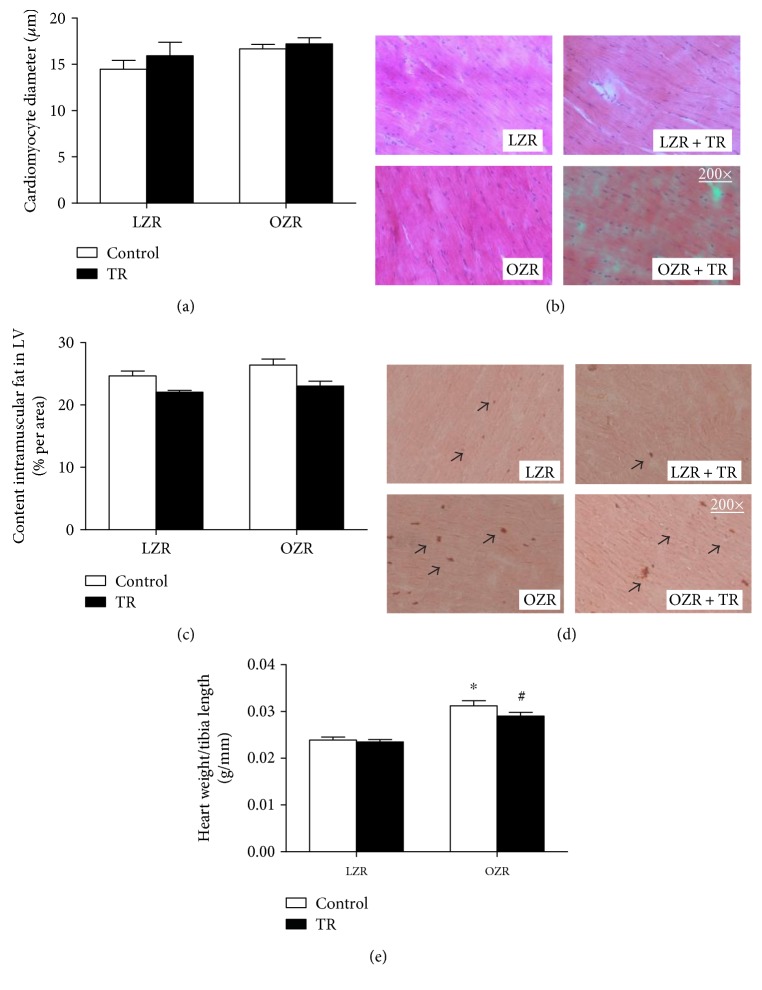
Effects of AET and obesity on cardiac intramuscular fat content and cardiomyocyte diameter. Cardiomyocyte (CMO) diameter (a). Representative histological images stained with hematoxylin and eosin (b). Cardiac intramuscular fat contents (c) were evaluated by histological analysis in LZR (lean group control), LZR + TR (lean trained group), OZR (obese group control), and OZR + TR (obese trained group). (d) Representative histological images stained with oil red for intramuscular fat. Arrows indicate fat red staining. (e) Total heart weight corrected by tibia length in LZR (lean group control), LZR + TR (lean trained group), OZR (obese group control), and OZR + TR (obese trained group). ^†^*p* < 0.005* versus* LZR + TR, ^&^*p* < 0.05 versus OZR + TR ^∗^*p* < 0.0001 versus LZR and LZR + TR, and ^#^*p* < 0.001 versus LZR and LZR + TR.

A previous study from our group showed pathological CH in OZR observed by echocardiography and LV mass/TL ratio [1]. Corroborating these data, we showed that the HW/TL ratio (mg/mm) was increased 29% in the OZR group compared with the LZR group, and the AET training decreased (8%) in the OZR + TR group (Figure 2(c)).

We assessed the LV intramuscular fat content and CMO diameter by histological analysis. LV intramuscular fat was increased in OZR compared with the trained groups (LZR + TR and OZR + TR) and decreased in LZR + TR compared with the LZR group (Figure 2(b)). Curiously, there were no significant differences in CMO diameter among the groups (LZR 13.8 ± 2.7 *μ*m; LZR + TR+ 17.7 ± 2.1 *μ*m OZR 17.1 ± 0.9 *μ*m; OZR + TR 18.2 ± 1.1 *μ*m) (Figure 2(a)). However, AET was effective in counteracting obesity-induced cardiac remodeling.

### 3.3. Molecular Markers of Pathological Cardiac Hypertrophy

**Figure 3:**
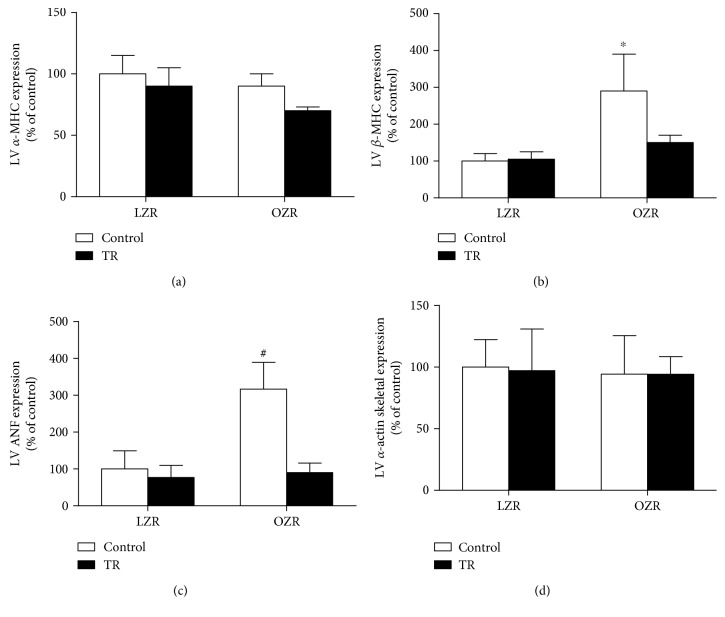
Effects of AET and obesity on *α*-MHC and *β*-MHC (alpha/beta-myosin heavy chain) (a, b), ANF (atrial natriuretic factor) (c), and *α*-actin skeletal (d) ratio in rat ventricles. Data are reported as means of 6 and SEMs of 5 animals in each group. ^∗^*p* < 0.05 versus LZR and ^#^*p* < 0.03 versus LZR, LZR + TR, and OZR + TR.

Pathological cardiac remodeling induces the expression of genes commonly expressed only in the fetal period such ANF, skeletal *α*-actin, and *β*-MHC (Figure 3()). The results of this study showed that obesity associated with increased *β*-MHC was increased in the OZR group compared with LZR, LZR + TR, and OZR + TR (Figure 3(b)). Similarly, ANF gene expression and swimming training were able to counteract it when compared with OZR + TR (Figure 3(c)). The results of this study showed that obesity and/or swimming training did not modify *α*-MHC gene expression (Figure 3(a)).

**Figure 4:**
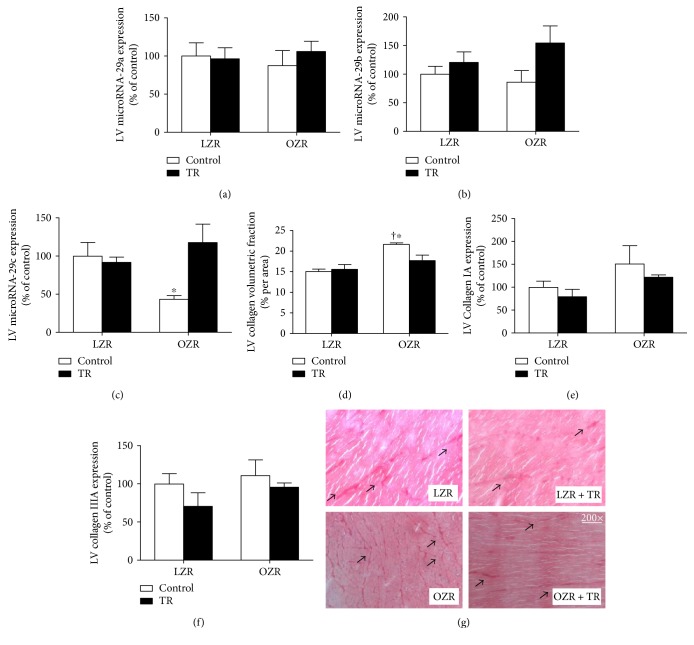
Effects of AET and obesity on cardiac microRNA-29 family expression, interstitial collagen volumetric fraction (CVF), and collagen expression. Cardiac microRNA-29a, microRNA-29b, and microRNA-29c expressions were evaluated by real-time PCR (a–c). LV interstitial CVF was evaluated by histological analysis, staining with Picrosirius red (d). Left ventricle (LV) collagen IA (COLIA) and IIIA (COLIIIA) gene expression was evaluated by real-time PCR (e, f). Representative histological images stained with Picrosirius red for CVF. Arrows indicate collagen red staining (g). LZR (lean group control), LZR + TR (lean trained group), OZR (obese group control), and OZR + TR (obese trained group). ^∗^*p* < 0.05 versus OZR + TR and ^†^*p* < 0.01 versus LZR and LZR + TR.

To confirm the involvement of obesity-regulated microRNAs in pathological CH, we analyzed the cardiac microRNA-29 family (microRNA-29a, microRNA-29b and microRNA-29c), whose expression affects collagen content. MicroRNA-29c expression was decreased in the OZR group compared with LZR, LZR + TR, and OZR + TR. AET resulted in microRNA-29c expression in the OZR + TR group approaching control levels (LZR: 100 ± 16.2%; LZR + TR: 92 ± 6.1%; OZR: 43 ± 4.7%; and OZR + TR: 118 ± 24.2%) (Figure 4(c)). The LV interstitial collagen volumetric fraction (CVF) was inversely proportional to the microRNA-29c expression level. These results show that CVF was increased in the OZR group compared with the LZR group. Interestingly, AET counteracted cardiac fibrosis in obesity, normalizing the CVF in the OZR-TR group (Figure 4(b)). However, gene expression of collagen IA and collagen IIIA did not change among the groups (Figures 4(c) and 4(d)).

### 3.4. Cardiac MicroRNA-1 and Calcium Signaling Proteins

**Figure 5:**
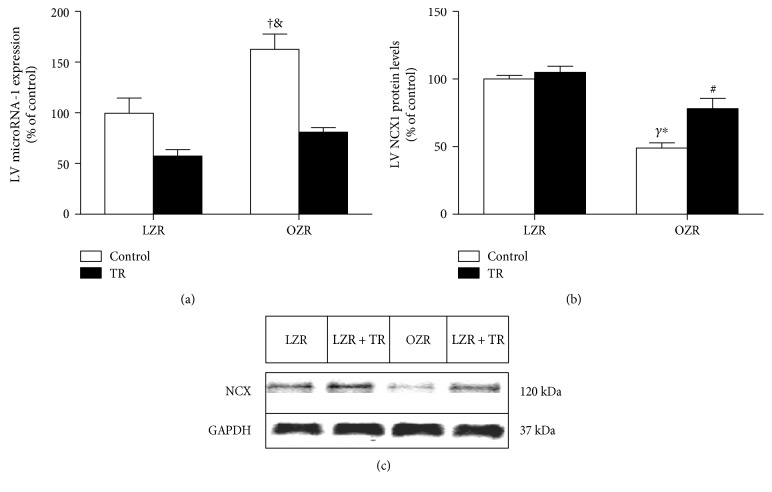
Effects of AET and obesity on microRNA-1 expression analyzed by real-time PCR (a) and NCX protein levels analyzed by western blot (b) in the left ventricle (LV). (c) Representative blots of NCX1 and GAPDH in LZR (lean group control), LZR + TR (lean trained group), OZR (obese group control), and OZR + TR (obese trained group). ^#^*p* < 0.05 versus LZR, ^†^*p* < 0.01 versus LZR, ^&^*p* < 0.001 versus LZR + TR and OZR + TR, ^Υ^*p* < 0.0001 versus LZR and LZR + TR, and ^∗^*p* < 0.05 versus OZR + TR.

MicroRNA-1 targets the NCX1 gene that is one of the most important cellular mechanisms for Ca^2+^ removal. MicroRNA-1 expression was increased in the OZR group compared with LZR, LZR + TR, and OZR + TR. Interestingly, AET was able to normalize microRNA-1 levels in the OZR + TR group. In addition, AET reduced microRNA-1 expression in LZR + TR compared with the LZR and OZR groups (Figure 5(a)). In parallel with the microRNA-1 expression, NCX1 expression was significantly reduced in the OZR group compared with LZR, LZR + TR, and OZR + TR. However, AET restored NCX1 expression in the OZR-TR group toward control levels (Figure 5(b)). The representative protein level by western blot is shown in Figure 5(c). These results show that NCX1 expression in obesity-induced pathological CR could be possibly reduced via increasing microRNA-1 expression by exercise training.

**Figure 6:**
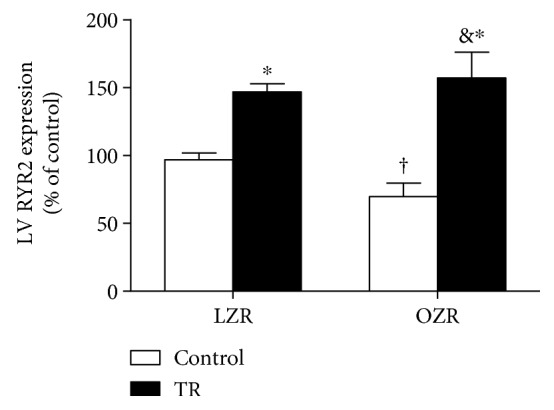
Ryanodine receptor 2 (RYR2) gene expression was evaluated by real-time PCR in the left ventricle (LV). LZR (lean group control), LZR + TR (lean trained group), OZR (obese group control), and OZR + TR (obese trained group). ^†^*p* < 0.01 versus LZR + TR, ^∗^*p* < 0.05 versus LZR, and ^&^*p* < 0.001 versus OZR.

**Figure 7:**
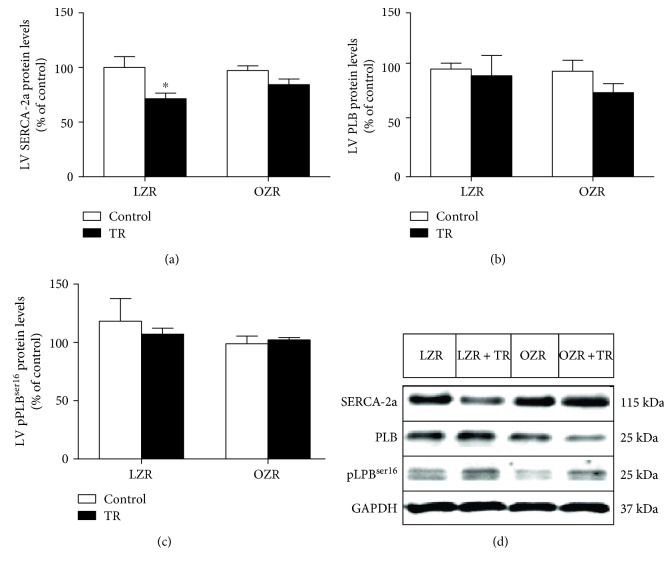
Effects of AET and obesity on calcium signaling proteins. SERCA-2a (a), phospholamban (PLB) (b), and phospholamban phosphorylated on serine16 (pPLB^ser16^) (c) protein levels were evaluated by probing western blots of left ventricle (LV) proteins. (d) Representative blots of SERCA-2a, PLB, pPLB^ser16^, and GAPDH in LZR (lean group control), LZR + TR (lean trained group), OZR (obese group control), and OZR + TR (obese trained group). ^∗^*p* < 0.05 versus OZR and LZR.

Other components of the calcium signaling pathway were also evaluated. Ryanodine (RYR2) gene expression increased in both groups (OZR + TR: 57 ± 5%; LZR + TR: 47 ± 19%) compared to the control group LZR (100 ± 9%) and also when compared with OZR (Figure 6()).  Figure 7(a) shows that SERCA-2a protein levels were decreased in the LZR + TR group compared with the LZR group; there were no significant differences in PLB and pPLB^ser16^ protein levels among the groups (Figures 7(b) and 7(c)). The representative protein level by western blot is shown in Figure 7(d).

## 4. Discussion

Obesity is a chronic disease that results from a convergence of genetic, psychological, and social factors. It is a risk factor for the development of cancer, diabetes, and cardiovascular diseases that induce pathological CH [2, 4, 29, 30]. This study evaluated molecular mechanisms of pathological cardiac remodeling induced by obesity and investigated whether AET reverses and/or prevents cardiac remodeling. Our results show that obesity-induced pathological cardiac remodeling leads to an increase in cardiac pathological hypertrophy markers and downregulation of microRNA-29c expression, which can be associated with the increase in the LV collagen volumetric fraction. In addition, obesity upregulated microRNA-1, which targets NCX1. NCX1 was decreased in the OZR group. In contrast, AET restored the pathological expression of microRNA-1 and microRNA-29c and their target genes, which likely counteracted the pathological cardiac remodeling and cardiac dysfunction in obesity.

As shown in a previous study from our group, AET was efficient in producing cardiovascular changes in OZR, such as a reduction in heart rate due to vagal hypertonia in the trained groups [4]. Barretti et al. [4] demonstrated that obesity leads to increased LV mass in OZR and that AET prevents this increase. Soci et al. [24] demonstrated that different intensities of swimming training lead to different magnitudes in the expression of microRNA-29c levels. Animals trained on the same protocol as the current study showed that the microRNA-29c levels decreased by 52% and that COLIAI and COLIIIAI expressions decreased by 27% and 38%, respectively. Animals trained on a higher intensity protocol presented an increase of 123% in microRNA-29c expression and decreases of 33% and 48% for COLIAI and COLIIIAI, respectively [24].

In the present study, as expected, AET was effective in decreasing epididymal and retroperitoneal fat. The OZR + TR group had a lower body fat content than the OZR group, as also shown by Disanzo and You who found that obesity led to an increase in endothelial growth factor A (VEGF-A) that is responsible for stimulating angiogenesis in adipose tissue counteracting glycolytic metabolism in this tissue and contributing to their decrease by exercise [31].

The OZR group presented pathological CH [4]. We quantified the CMO diameter; however, there were no differences among the groups, which suggest that the increase in cardiac mass in the OZR group is due to increased LV intramuscular fat and/or cardiac collagen. Moreover, to corroborate with the pathological cardiac hypertrophy phenotype, obesity induced an increase of fetal gene expressions, such as ANF and *β*-MHC.

Here, we showed that LV intramuscular fat was higher in the OZR group compared with LZR, LZR + TR, and OZR + TR. In fact, the reduced cardiac fat in the OZR-TR group caused by AET can be explained as part of the 13% reduction in LV mass or even the 7% reduction in total heart weight compared with that in OZR. Some studies suggest that this increased fat content in the myocardium leads to heart dysfunction and predisposition to chronic diseases [32, 33]. These findings reinforce the importance of AET as a preventive tool against cardiovascular pathologies.

**Figure 8:**
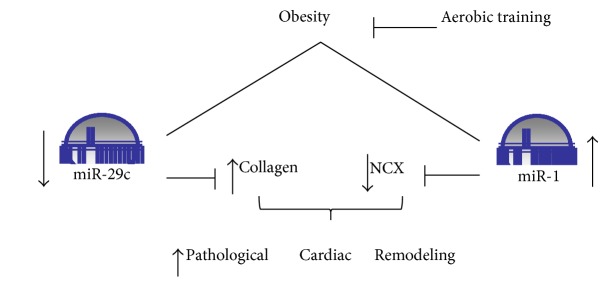
Schematic representation of the effects of AET on obesity-induced pathological cardiac remodeling via the involvement of microRNA-1 and microRNA-29c. AET is a powerful stimulus modulating microRNA-1 and microRNA-29c that regulates their target genes (NCX1 and collagen), thereby counteracting the pathological CH phenotype.

The microRNA-29 family has been described to negatively regulate collagen content and to be highly responsive to AET [22, 24, 25]. Studies have shown that AET increases microRNA-29 expression in the heart and consequently decreases collagen expression and protein levels [24, 25]. In the present study, obesity decreased the cardiac microRNA-29c expression in OZR by 47% compared with LZR, which induced an increase in the cardiac CVF. Thus, AET was able to normalize cardiac microRNA-29c expression and CVF in OZR + TR, and these results suggest that AET has a cardioprotective effect against pathological CH as shown in Figure 8.

In our previous study, although there was no statistical difference (*p* = 0.07), a 25% reduced time E/A wave ratio was found when OZR was compared with untrained LZR [4], suggesting damage in the contractile myocardium. In the present study, we demonstrated that the cardiac collagen content in OZR could induce impaired compliance. Dong et al. [12] showed reduced compliance in isolated CMO from obese mice. In our study, we investigated molecular mechanisms involved in diastolic dysfunction induced by obesity. We evaluated the levels of calcium transporter proteins involved in contractile mechanisms. MicroRNA-1 targets the NCX1 protein and is an important regulator of calcium mechanisms in the heart [26]. MicroRNA-1 was significantly increased in OZR compared with LZR, LZR + TR, and OZR + TR, in contrast to previous studies that have shown a reduction in microRNA-1 in pathological cardiac remodeling caused by others pathologies [22, 24, 26]. Thus, we hypothesized that cardiac remodeling induced by obesity is a milder compensatory response than that found in other pathologies, such as CH due to ischemic diseases [22]. Interestingly, AET caused downregulation of microRNA-1 expression in OZR + TR compared with OZR, showing that it could be an important tool against the pathological phenotype caused by obesity. AET was also able to reduce the expression of microRNA-1 in LZR + TR compared with LZR, data that reinforces the profile observed in the previous AET studies [24].

The NCX1 protein, which is the direct target of microRNA-1, was downregulated in OZR compared with the other groups (LZR, LZR + TR, and OZR + TR); this data indicates a possible antagonism between NCX1 and microRNA-1 expressions [34]. In contrast, AET was effective in restoring NCX1 levels in OZR + TR compared with OZR.

In the present study, there was an increase in the RYR2 receptor expression in both trained groups (LZR + TR and OZR + TR) compared with their controls (LZR and OZR). Our findings were different from those found in a study with rats submitted to AET and food restriction, where no significant change in RYR2 receptor expression was found [35, 36]. This could be because the swimming training was most effective to promote this adaptation in obesity phenotype. Increased RYR2 receptor expression improves the release of sarcoplasmic Ca^2+^, which could lead improvements in cardiac contractility [20, 34–36].

We also observed that SERCA-2a expression was decreased in LZR + TR compared with LZR and a tendency in OZR + TR compared with OZR. While SERCA-2a expression decreased, the RYR2 expression was increased that could be causing an imbalance in the sarcoplasmic Ca^2+^ content. However, it is known that SERCA-2a function is dependent on the phosphorylation of the PLB protein [11] and there were no differences in total PLB and pPLB^ser16^ expressions. Thus, NCX1 could be contributing to maintain intracellular normal Ca^2+^ concentration, at least in OZR + TR compared with OZR, in these trained animal models. These findings and the results concerning the upregulation of microRNA-1 can be associated with the downregulation of NCX1 in OZR which suggest that cardiac contractile dysfunction was prevented in OZR + TR improving these mechanisms, thus improving the cardiac function [4].

Our study demonstrates for the first time that AET was efficient in restoring the microRNA-1 and microRNA-29c to nonpathological levels in obesity, as well as its targets NCX1 and collagen, respectively.

Despite the strong association between microRNAs and their target genes, we do not demonstrate a direct proof of concept between them. However, the genes were validated to these microRNAs by other authors [34, 37]. Thus, further studies are needed to assess whether modulation of the microRNA-1 and microRNA-29c in vivo in the obesity phenotype would play a key role in preventing pathologic cardiac remodeling.

In conclusion, obesity downregulated microRNA-29c in OZR possibly leading to increased cardiac collagen content. Conversely, microRNA-1 levels were upregulated, and their target gene NCX1 was decreased in OZR, maybe causing diastolic dysfunction in these animals as we showed before [4]. Figure 8 shows a schematic representation of these data. One implication of our findings is that AET protects the heart against an aberrant increase of extracellular matrix components and prevents calcium-signaling pathway dysfunction in the cardiac remodeling phenotype caused by obesity through microRNA modulation.
