# Corrigendum to “Exercise Training Restores Cardiac MicroRNA-1 and MicroRNA-29c to Nonpathological Levels in Obese Rats”

**DOI:** 10.1155/2018/4818310

**Published:** 2018-02-25

**Authors:** André C. Silveira, Tiago Fernandes, Úrsula P. R. Soci, João L. P. Gomes, Diego L. Barretti, Glória G. F. Mota, Carlos Eduardo Negrão, Edilamar M. Oliveira

**Affiliations:** ^1^Laboratory of the Biochemistry and Molecular Biology of Exercise, School of Physical Education and Sport, University of Sao Paulo, Sao Paulo, SP, Brazil; ^2^Heart Institute (InCor), Medical School, University of São Paulo, São Paulo, SP, Brazil

In the article titled “Exercise Training Restores Cardiac MicroRNA-1 and MicroRNA-29c to Nonpathological Levels in Obese Rats” [1], an acknowledgment should be added as follows.

This study was supported by Fundação de Amparo à Pesquisa do Estado de São Paulo (FAPESP—2009/18370-3 and 2010/50048-1); Programa de Inovação Tecnológica/CEPID (Centros de Pesquisa, Inovação e Difusão) (2013/07607-8); USP/PRP: Núcleo de Apoio à Pesquisa com microRNAs-NAP and Pro-Infra; Conselho Nacional de Desenvolvimento Científico e Tecnológico-CNPq (no. 308267/2013-3); Programa PIBIC; and the Brazilian Federal Agency for Support and Evaluation of Graduate Education (CAPES-PROEX).
